# Microsurgical treatment of lumbar paravertebral tumors via lateral retroperitoneal approach: operative technique and a series of 6 patients

**DOI:** 10.1186/s12893-022-01774-x

**Published:** 2022-08-27

**Authors:** Lei Zhang, Shichao Chen, Kai Wang, Hao Wu

**Affiliations:** 1 Department of Neurosurgery, Xuanwu Hospital Capital Medical University, 45 Changchun Street, 45 Changchun Street, Beijing, People’s Republic of China 100053; 2 Department of Neurosurgery, Capital Medical University Affiliated Beijing Ditan Hospital, 8 Jingshundong Street, Beijing, People’s Republic of China 100015

**Keywords:** Lateral retroperitoneal approach, Lumbar paravertebral tumors, Microsurgical treatment

## Abstract

**Objective:**

To investigate the surgical techniques and postoperative therapeutic effectiveness of microsurgical treatment of lumbar paravertebral tumors via lateral retroperitoneal approach.

**Methods:**

The clinical data of 6 cases with lumbar paravertebral tumors treated by lateral retroperitoneal approach in the Neurosurgery department of Xuanwu Hospital, Capital Medical University were analyzed retrospectively. The mean operation time, blood loss, incision length, length of hospital stay, and the resection rate of paravertebral tumors were collected, and the score of The Ability to Perform Activities of Daily Living (ADL) and incidence of postoperative complications was recorded.

**Results:**

The operation time ranged from 56 to 181 min, with an average of (94.8 ± 48.3) minutes. The blood loss was between 5 and 100 ml, with an average of (31.7 ± 37.5) ml. The incision length was 6–7 cm, with an average of (6.7 ± 0.5) cm. The hospitalization length was between 5 and 11 days, with an average of (8.7 ± 2.6) days. The resection rate of paravertebral tumors was 100%. Postoperative pathological diagnosis results revealed 4 cases of schwannoma, 1 case of ganglioneuroma, and 1 case of malignant small round cell tumor. During the 3-month follow-up, there were no tumor recurrence, abdominal infection, incision infection, incisional hernia, or death, and there was no significant decrease in the ADL score compared with that before the operation.

**Conclusion:**

The surgical treatment of lumbar paravertebral tumors via the lateral retroperitoneal approach has the advantages of the short operation time, minimally invasive procedures, quick postoperative recovery, and fewer complications.

## Introduction

Lumbar paravertebral tumors in the retroperitoneal space are mainly neurogenic tumors, most of which are schwannomas, accounting for 0.7–2.7% of retroperitoneal tumors [1, 2], and mostly originate from the dorsal root of the spinal nerve. Due to the loose structure of the posterior peritoneal lumbar paravertebral tissue and the sizeable intraperitoneal space, the tumor grows to a large size without apparent symptoms. The treatment for paravertebral tumors is mainly focused on surgery [3–6]. Conventional surgical methods include the standard posterior midline approach, which requires separation or transverse dissection of the paraspinal muscles to gain more surgical exposure, and even lamina and facet resection to restore spinal stability after tumor removal. Another method is to remove the tumor through an anterior approach [4, 6, 7]. Wiltse et al. proposed an approach through the paraspinal intermuscular space by first separating the space between the longissimus and the multifidus to approach the lateral side of the transverse process, then removing the intertransverse ligament to expose the paravertebral space, so that the tumor can be removed [8]. Traditional surgical methods have long operation time, traumatic surgical incisions, heavy blood loss, and prolonged hospitalization. In recent years, removing retroperitoneal tumors through laparoscopic surgery is a relatively minimally invasive surgical method, but common paravertebral tumors are adjacent to the aorta, inferior vena cava, and other organs. These difficulties bring high risk to laparoscopic surgery [4, 9–16]. With the introduction of the OLIF (oblique 1umbar interbody fusion, OLIF) surgical technique in 2012 and the development and innovation of this surgical technique by scholars and surgeons [17, 18], the minimally invasive surgical technique can reach the specific surgical area via lateral retroperitoneal approach. It has the advantages of safety, effectiveness, and less blood loss while preventing damage to spine stability. From June 2019 to December 2020, Xuanwu Hospital of Capital Medical University performed surgical treatment for six lumbar paravertebral tumors via a lateral retroperitoneal approach. All came out with satisfactory clinical results, which are reported and summarized as follows.

## Methods

A retrospective review of all cases of lumbar paravertebral tumors that underwent surgical treatment via lateral retroperitoneal approach at our institution between from June 2019 to December 2020 was performed.

### Clinical data

Inclusion criteria:

i. The paravertebral tumor is located outside the spinal canal, behind the peritoneum, and there is no space-occupying tissue (tumor) in the intervertebral foramen.
ii. The location of the tumor corresponds to the lumbar spine segments.
iii. Complete follow-up data.

Exclusion criteria:

i. Tumors in the lumbar intervertebral foramen or the spinal canal.
ii. The tumor's location corresponds to higher or lower spine segments such as the thoracic or sacral spine.
iii. Existence of systemic diseases that cannot tolerate surgical treatment, such as coagulation dysfunction.

### Radiological imaging data

**Fig. 1:**
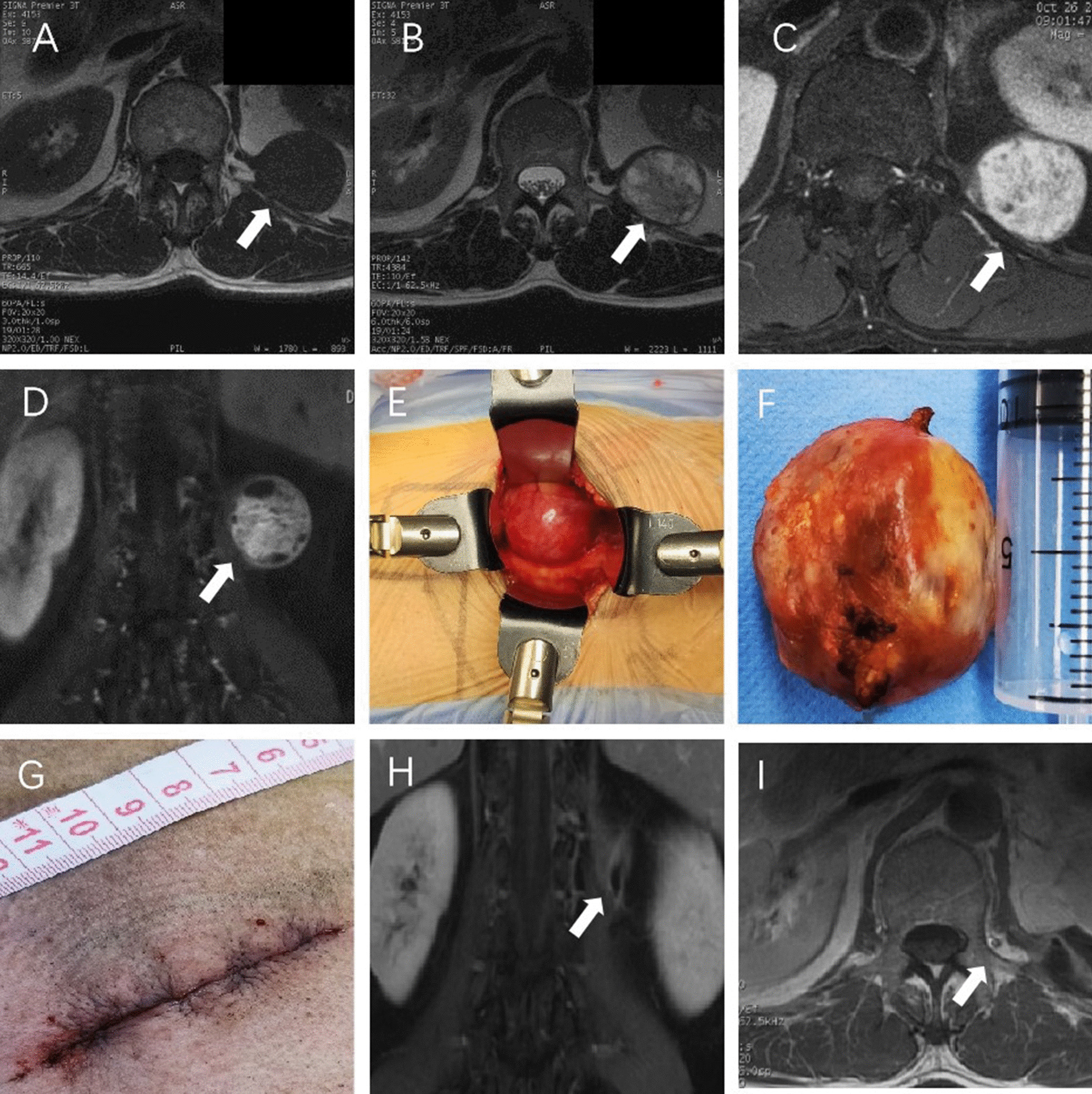
Microsurgical treatment of lumbar paravertebral tumors via lateral retroperitoneal approach. **A** MRI T1-weighted: a round-like paravertebral tumor (white arrow)at the posterior side of the kidney in the retroperitoneum, low signal. **B** MRI T2-weighted: small amount of mixed and uneven high signal, round-like tumor (white arrow). **C** MRI Gad-enhanced T1-weighted: axial view, a round-like tumor (white arrow) posterior to the kidney can be observed, significantly enhanced. **D** MRI Gad-enhanced T1-weighted: coronal view, a round-like tumor (white arrow) can be observed at L1–2 vertebral body level. **E** Intraoperative image: a 14 cm deep retractor fully exposes the tumor after retraction. **F** Postoperative image: The tumor was completely resected along the inner surface of the capsule, about 4 cm in diameter. **G** Postoperative image: the surgical incision length is about 7 cm. **H**, **I** Postoperative MRI image: image of retroperitoneal space, complete removal of the tumor (white arrow) located posterior to the kidney

All patients in this group underwent X-Ray, CT, and enhanced MRI examinations. A General X-Ray examination confirmed no enlargement of the intervertebral foramen and no spinal deformity or other manifestations. CT examination utilized thin-layer slices scanning and three-dimensional reconstruction was conducted to clarify the relationship between the tumor and the bony structures, whether there was an expression of bony structure erosion or other manifestations. At the same time, CT examination provided imaging evidence for evaluating spine stability. Enhanced magnetic resonance imaging examination revealed that localized para-lumbar spine mass was located outside the spinal canal and behind the peritoneum. The T1-weighted image showed equal or lower inhomogeneous signal (Fig 1A), the T2-weighted image showed equal or slightly higher inhomogeneous signal (Fig 1B), and the gad-enhanced image showed mild inhomogeneous enhancement, clear boundary, and no tumor in the intervertebral foramen (Fig 1C, D).

### Surgical methods

This group of cases is operated all by the same surgeon. After general anesthesia tracheal intubation, take the lateral position (the affected side facing upward), the operating table was adjusted to jackknife position, and the iliac crest and intercostal space on the affected side are fully extended. The projection of lumbar intervertebral space in interest on the lateral side of the skin is acquired and located with the C-arm imaging system. A straight incision on the skin started from the projection of the anterior edge of the vertebral body that went anteriorly parallel to the external oblique muscle was made (Fig 1G), the skin and subcutaneous tissue were cut open (cut off the ribs or spread the intercostal space with spreaders if necessary), muscles of the abdominal wall was separated layer by layer along the muscle bundle direction of the external oblique muscle, internal oblique muscle, and transversus abdominis muscles, blunt dissection with fingers was made to separate retroperitoneum, under the condition where kidney, ureter, peritoneum, intestine and other abdominal structures were fully protected, the peritoneum was retracted to the ventral side with retractors, exposing the retroperitoneal space (Fig 1E). The deep retroperitoneal paravertebral space was gradually separated by the dissector, fully exposing the tumor. The relationship between the tumor and surrounding tissues, especially blood vessels and nerves, was distinguished and analyzed. If the tumor was encapsulated, the capsule was cut open with a scalpel, the tumor was separated along the inner wall of the capsule, the proximal and distal nerve connections were cut off, and the tumor was removed entirely (Fig 1F). If the tumor is relatively large, it can be removed in pieces. Bleeding was completely stopped in the residual cavity, the retractors were removed, and the transversus abdominis, internal oblique muscle, external oblique muscle, subcutaneous tissue, and skin were sutured layer by layer.

### Outcomes and follow-up

In this study, the operation time (the time from the start of skin incision to the end of skin suturing), the amount of blood loss, the length of the surgical incision, and the length of the hospital stay (the time from admission to discharge) were selected as the observation indicators; the effectiveness of tumor resection was evaluated by comparing preoperative and postoperative enhanced MRI; At follow-up, the scoring method of Barthel Index was used to score the patient's ability of daily living activities. At the same time, the follow-ups also include the level of patient's clinical symptom relief, whether there is an abdominal infection, incisional infection, incisional hernia, or death.

This case series has been reported in line with the PROCESS Guideline.

## Results

### General information

All the six patients in this group were male, aged between 19 and 66 years old, with an average of (42.2 ± 19.4) years old. The detailed clinical data of these patients are shown on Table 1. Corresponding segments for paravertebral tumors were located para T12–L1 in 1 case, L1–L2 in 1 case, L1–L3 in 2 cases, and L5–S1 in 2 cases. There were 4 cases on the left side and 2 cases on the right side. 2 cases of patients were found during asymptomatic physical examination, 2 cases of patients were found through symptoms of back pain, 1 case of a patient was found through symptoms of hip pain, and 1 case of a patient was found through symptoms of pain in the left waist and calf. ADL scores preoperatively were 100 for every patient (Table 2). Postoperative pathological diagnosis results revealed 4 cases of schwannoma, 1 case of ganglioneuroma, and 1 case of malignant small round cell tumor.

**Table 1:** Patient demographic characteristics and presentation

Case no.	Age (year)	Sex	Location	Symptomatology	Pathology
1	64	M	L1–L2	None	Schwannoma
2	29	M	T12–L1	None	Schwannoma
3	44	M	L1–3	Low back pain	Schwannoma
4	66	M	L5–S1	Low back pain	Schwannoma
5	19	M	L5–S1	Left waist and calf pain	Ganglioneuroma
6	31	M	L1–3	Hip pain	Malignant small round cell tumor

*M* male, *T* thoracic, *L* lumbar, *S* sacral

**Table 2:** ADL score of patients in perioperative period and follow-up

The ability to perform activities of daily living (ADL) score			
	Preoperative	Postoperative	Follow-up after 3 months
Patient 1	100	90	100
Patient 2	100	90	100
Patient 3	100	85	95
Patient 4	100	65	90
Patient 5	100	100	100
Patient 6	100	95	100

### Operation results

The operation time ranged from 56 to 181 min, with an average of (94.8 ± 48.3) min. The volume of blood loss was between 5 and 100 ml, with an average of (31.7 ± 37.5) ml. The surgical incision length was 6–7cm, with an average of (6.7 ± 0.5) cm. The hospitalization length was between 5 and 11 days, with an average of (8.7 ± 2.6) days. The resection rate of paravertebral tumors was 100%, indicated by postoperative enhanced magnetic resonance imaging re-examination.

The patients' earliest symptom of back pain was relieved entirely postoperatively, and patients with hip pain, left waist, and calf pain reported symptom relief after surgeries.

### Follow-up results and complications

Enhanced magnetic resonance imaging was performed postoperatively during follow-ups for all six patients. There was no recurrence of tumors, no abdominal cavity infection and no incisional infection, no incisional hernia, and no death cases (Fig 1H, I). The patient with a malignant small round cell tumor underwent chemotherapy and radiotherapy after surgery. The follow-up conducted after three months of surgeries revealed no significant decrease in the ADL score compared to that before the operation (Table 2). All six patients were able to perform daily tasks and self-care. Only one patient still experienced mild pain around the surgical incision.

## Discussion

Lumbar paravertebral tumors in the retroperitoneal space are mainly neurogenic tumors, and schwannomas are very common among them. In this group of cases, 5 cases are neurogenic tumors, of which 4 are schwannomas [1, 2]. Due to the loose tissue structure of the retroperitoneum, the tumor was not noticed until it was significant in size. Most patients had no obvious or specific symptoms. However, some patients with schwannoma had nerve root symptoms. Among the cases in this group, there were 2 asymptomatic patients, 2 patients with lower back pain, 1 patient with hip pain, and 1 patient with left waist and calf pain [3–6]. Surgical treatments are usually required [6, 9]. Due to the low incidence of lumbar paravertebral retroperitoneal tumors and relatively slow improvement of traditional surgical instruments, the surgical treatment methods are limited. Traditional invasive open surgeries or trans-muscular tumorectomy via posterior approach are more traumatic to patients [4, 6, 7]. With the progress of surgical instruments, the deepening of minimally invasive surgery, and the improvement of surgeons' surgical techniques. Laparoscopy has been gradually applied to treating retroperitoneal tumors under the condition of ideal surgical location and volume [9]. In addition, when the tumor is located near the vertebra, large blood vessels, or organs, it often requires a more meticulous operation, increasing the risk of surgery [4, 9]. Intraoperative neurophysiological monitoring (IONM) was not performed in this series. Nevertheless, we believe that IONM is essential in the cases of tumor encapsulating nerve roots.

Due to the emergence of the minimally invasive approach through the lateral retroperitoneal space, surgeons can treat lumbar paravertebral tumors with minimally invasive approaches. This approach can achieve the same minimally invasive purpose as laparoscopic surgery. Besides, it can also safely treat paravertebral tumors adjacent to large vessels and surrounding complex anatomical structures. All patients in this group were treated with a lateral retroperitoneal approach to remove lumbar paravertebral tumors. The average operation time in this group was (94.8 ± 48.3) min, the average blood loss was (31.7 ± 37.5) ml, and the average hospital stay was (8.7 ± 2.6) days. No complications such as abdominal cavity infection, incisional infection, or incisional hernia were observed. Compared with traditional surgical methods, the microsurgical treatment of lumbar paravertebral tumors via the lateral retroperitoneal approach has the advantages of minor trauma, shorter operation time, and less blood loss [4, 6, 7].

### Treatment method

This study applied microsurgical treatment of lumbar paravertebral tumors through the lateral retroperitoneal approach. Several considerations are as follows:

i. The tumor can be directly removed through the minimally invasive lateral retroperitoneal approach, and taking advantage of surgical operation in the natural interstitial space can reduce damage to surrounding tissues compared with trans-abdominal or trans-muscular approaches.
ii. The surgical trauma from performing lateral abdominal wall incision is smaller than that of trans-abdominal and posterior trans-muscular tissue separation, which benefits the patient's postoperative recovery.
iii. Through the lateral retroperitoneal approach, operating under direct view with the help of a surgical microscope, the surgical field is clear, and the operating space is sufficient. Operations are not affected by the position or volume of the tumor. Also, it can help avoid passing through major blood vessels and essential nerve structures, thus reducing the corresponding damages and complications. At the same time, proper utilization of surgical instruments by surgeons can reduce the retraction and damage to the surrounding tissue, avoiding conditions such as ureteral damage, sympathetic nerve injury, lumbar plexus injury.
iv. The lateral approach does not affect the stability of the spine, resulting in no need for internal fixation. It can reduce implant use and also reduces the cost of hospitalization.

### Indications for surgery

i. Paravertebral tumors of the lumbar spine. The highest tumor corresponding segment in this group of cases is the thoracic 12 spine segment. If it continues upward, due to the interference of the pleura, the surgical operation becomes more complex, and the risk of pleural damage is increased. If the position of the tumor is too low, the operation space gets limited due to the interference of the iliac crest. Also, the blood vessels are abundant anteriorly to the sacrum, which increases the risk of surgery [19].
ii. The spinal canal and intervertebral foramen are not affected by the tumor. If the intervertebral foramen and spinal canal are involved, excessive retraction during tumor resection may damage the spinal nerve root or cauda equina, leading to nerve function damage, which brings high risk.
iii. Retroperitoneal tumors. The retroperitoneal space is relatively broad and maneuverable.

### Essential technique

i. According to the specific position of the lumbar spine segments corresponding to the tumor, the tumor position is marked laterally on the skin under the C-arm imaging system, and the surgical incision is identified. The incision runs along the ribs. If the ribs are interfering with the incision, part of the ribs can be removed. It is also feasible to spread the intercostal space if the tumor is small.
ii. Retract the abdominal wall and other tissue around the tumor. Enter the retroperitoneal space and bluntly separate the retroperitoneal fat with fingers to avoid entering the peritoneum to avoid damaging the ureter and blood vessels [20, 21]. In particular, do not excessively retract the psoas major muscle and avoid irritating the psoas major and lumbar plexus, which may result in hip flexion weakness and deficiency postoperatively [22–25].
iii. The principles of tumor resection should be followed strictly, tumor capsules should be distinguished carefully, and the tumors should be removed completely.
iv. To close the incision of the abdominal wall, the transversus abdominis, internal oblique muscle, and external oblique muscle should be sutured tightly to avoid iatrogenic incisional hernia caused by the increased pressure in the abdominal cavity.

## Conclusion

Microsurgical treatment of lumbar paravertebral tumors via lateral retroperitoneal approach has the advantages of a short operation time, minimal invasiveness, quick recovery, and fewer complications. With the advancement and development of surgical techniques and instruments, this surgical method will be further promoted and applied in clinical practice.

## Data Availability

All data generated or analyzed during this study are available from the corresponding author on reasonable request.
